# Supplementary material for: Unveiling the impact of community knowledge in malaria programmes: A scoping review protocol
Source: PLoS One. 2024 Jul 5;19(7):e0306776. doi: 10.1371/journal.pone.0306776 (PMC11226004; doi:10.1371/journal.pone.0306776)
Supplement: S1 Fig — (PDF) [file pone.0306776.s002.pdf]

S1 Figure: PRISMA 2020 flow diagram for new systematic reviews which included searches of databases and registers only

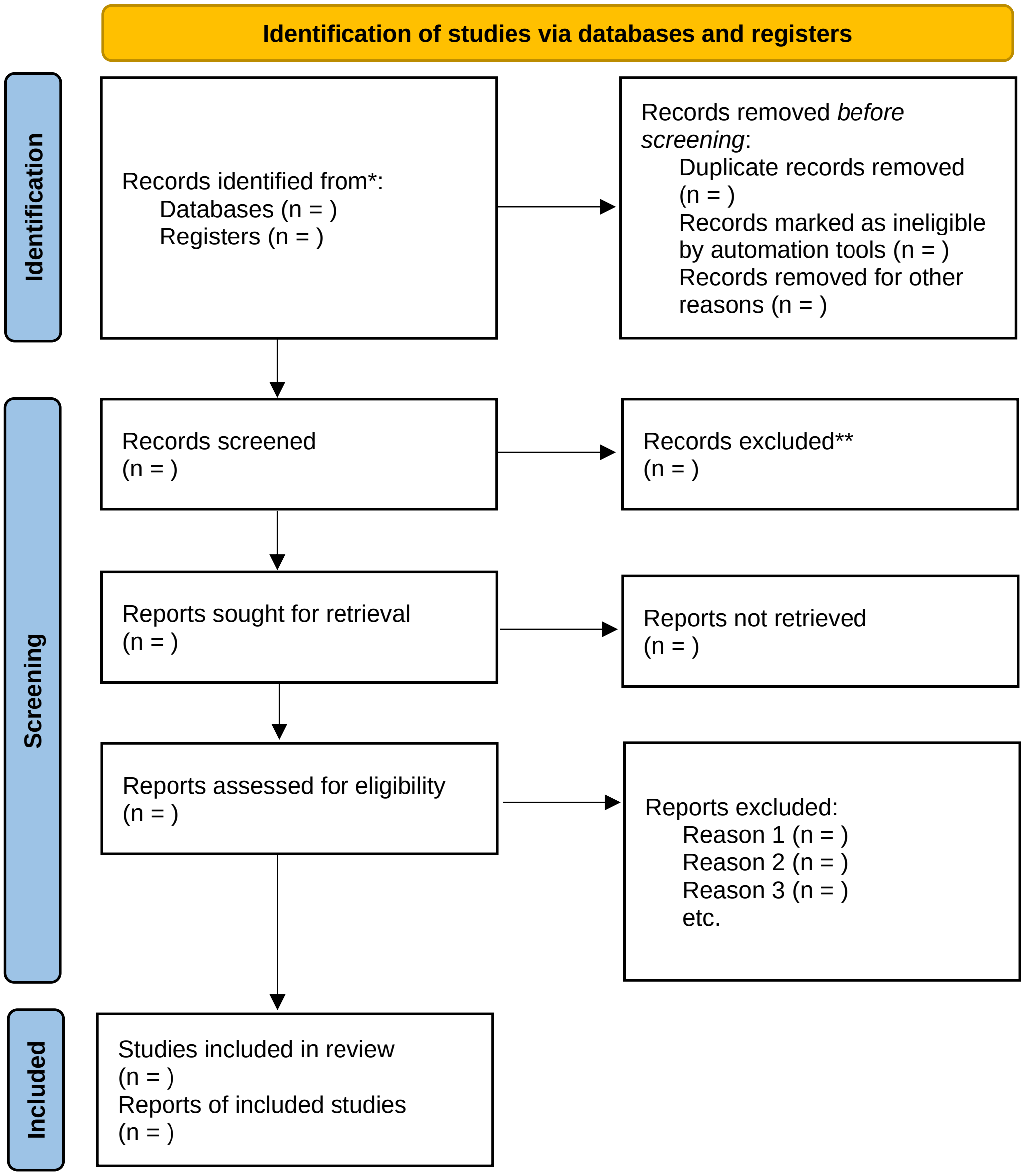

*Consider, if feasible to do so, reporting the number of records identified from each database or register searched (rather than the total number across all databases/registers).

**If automation tools were used, indicate how many records were excluded by a human and how many were excluded by automation tools.

From: Page MJ, McKenzie JE, Bossuyt PM, Boutron I, Hoffmann TC, Mulrow CD, et al. The PRISMA 2020 statement: an updated guideline for reporting systematic reviews. BMJ 2021;372:n71. doi: 10.1136/bmj.n71

For more information, visit: http://www.prisma-statement.org/
