# Supplementary material for: Unveiling the impact of community knowledge in malaria programmes: A scoping review protocol
Source: PLoS One. 2024 Jul 5;19(7):e0306776. doi: 10.1371/journal.pone.0306776 (PMC11226004; doi:10.1371/journal.pone.0306776)
Supplement: Attachment — Submitted filename: Response to Reviewers.docx [file pone.0306776.s003.docx]

| Reviewer Comments | Author responses |
| --- | --- |
| 1. Line 23: Please edit it to "extracting bibliographic details and outcome information" or add more domains to the sentence. | The revision has been made as instructed. **(refer to line 23)** |
| 2. Line 28-29: If the authors mention gaps, they need to strategize how to identify them in this review. Generally (without the presence of framework), a scoping review can find evidence on a particular topic, however, for finding gaps (and evidence both), evidence and gap maps are there. | The discussion section has strategically identified research gaps by emphasizing the advantages of integrating evidence and gap mapping methodologies into future malaria prevention and control. **(refer to lines 220-229)** |
| 3. Line 30: Please edit to "assist" policymakers and empowering others. A suggestion is to mention more implications, currently it is only to design an intervention. | The revision has been made as instructed. **(refer to lines 31-34)** |
| 4. Line 105: Please define, what is community knowledge of malaria | The definition of community knowledge of malaria was provided in the third paragraph of the introduction section. **(refer to lines 55-57)** |
| 5. Line 108-109: Avoid using "these" in the objective. | The revision has been made as instructed. **(refer to line 114)** |
| 6. Line 130: Please edit to "Inclusion and exclusion criteria" or "Eligibility criteria" | The revision has been made as instructed. **(refer to line 135)** |
| 7. Line 138-140: The eligibility criteria does not give information about the modelling studies, or studies that may be based on simulation, prediction or machine learning. Please add information in inclusion or exclusion. A suggestion is to not repeat the sentences, eg: including English language articles implies non-English will be excluded. | Articles based on modelling, simulation, prediction, and machine learning will be excluded from this review. Therefore, the exclusion criteria section will explicitly outline these criteria. **(refer to line 155)**  Thank you for your feedback. We will carefully cross-check the content to ensure that no sentences are repeated |
| 8. Line 143: The exclusion can also be according to programs, will you exclude the dengue and chikungunya programs, also give information on what will you do if the study is about all mosquito borne diseases? | This review focuses exclusively on malaria. We have excluded other mosquito-borne diseases such as dengue and chikungunya due to differences in the vectors and their bionomics, which significantly impact malaria. Additionally, preventive and control measures for malaria differ from those for these two diseases. |
| 9. Line 144: Please edit, as systematic reviews are not secondary studies, you can write, reviews, eg: systematic reviews and other reviews. | The revision has been made as instructed. **(refer to line 149)** |
| 10. Line 148: Can you specify why multi-country studies will be excluded? It is an important evidence, however, if the authors have a specific reason, please list. | After further discussion, we have decided to include multi-country studies in our analysis. |
| 11. Line 149: Is there any specific reason why book chapters will be excluded, if the book chapter is about a primary study and fits your inclusion, will you exclude it? Abstracts and conference proceedings can be excluded because it is not peer reviewed. | Primary inclusion will prioritize eligible published articles sourced from selected databases. Book chapters will be excluded due to potential duplication of findings already present in the original published articles. |
| 12. Line 151: Is any search specialist involved in this review, if yes, please describe who will run the search? | The article search will be executed using carefully curated keywords tailored for this review. Experienced researchers specializing in malaria studies will oversee the search process. These keywords have been developed in collaboration with experts from the malaria programme at the Ministry of Health Malaysia. The search will be conducted by FAAR and AMMN across the designated databases. **(refer to lines 165-167)** |
| 13. Line 153: Please replace "scrutinised" with "searched" | The revision has been made as instructed. **(refer to line 158)** |
| 14. Line 161-162: Please examine reference lists of the final included articles, as the initial search will have many results and to go through each of their bibliographies will be a massive task and delay the review process. | The revision has been made as instructed. **(refer to lines 167-168)** |
| 15. Line 163-164: Please write for how many days will you wait if you do not receive a reply from the authors, a suggestion is to contact the corresponding author as it will be easy to find their email. | The revision has been made as instructed. **(refer to lines 168-170)** |
| 16. Line 165: Please mention if the authors will be using any digital applications (eg: Covidence or Rayyan or EPPI Reviewer) for the process of screening. If the authors will be using Excel spreadsheets, I request the authors to mention that as well. Please write few points about the process of compilation and deduplication in this section. | During the screening phase, article data will be stored in Microsoft Excel. Details regarding the compilation and deduplication process are outlined in the screening strategy section. |
| 17. Line 165: Please mention about the second level screening (full text screening) and how full texts of the articles will be accessed and screened (currently, the authors have only explained title and abstract screening). Please write what will be the procedure the authors will follow if full text (PDF) of the studies are not available. | The full text screening process and the procedure to be followed if articles are not available have been detailed in the screening strategy section. |
| 18. Line 165: A suggestion is, two authors independently screen each article and a third reviewer can be involved if they authors do not achieve consensus. You can assign one author to review all the excluded title and abstracts (if you want to improve quality further). According to the guidelines if two reviewers achieve consensus, it is adequate for inclusion and exclusion, and involving 4 would not improve the quality of the screening, but you will in turn require double the time. Please consider here, if you can give the required time, you can go ahead with the currently mentioned method. If you have some methods paper which states that quality of screening is better if higher number of reviewers are involved in screening the same set of articles, please provide reference. | The revision has been made by reducing the reviewers as instructed. **(refer to lines 174-176)** |
| 19. Line 172: A suggestion is to pilot the data extraction form with a few articles and edit the form before beginning with the final extraction. This will ensure the authors are able to extract what they intended to. | The revision has been made as instructed. **(refer to lines 193-195)** |
| 20. Line 185: Please use PRISMA 2020 flow diagram (Page et al., 2021) to depict the screening process. | The revision has been made as instructed. **(refer to line 199)** |
